# Supplementary material for: Modeling glioblastoma heterogeneity as a dynamic network of cell states
Source: Mol Syst Biol. 2021 Sep 16;17(9):e10105. doi: 10.15252/msb.202010105 (PMC8444284; doi:10.15252/msb.202010105)
Supplement: Supplementary file 6 — Source Data for Figure 5 [file MSB-17-e10105-s004.zip › Figure5A_sourcedata/GSEA_3017/nosigdb_stateB.GseaPreranked.1622795021725/FELDMAN_2020_G2-M.html]

Details for gene set FELDMAN\_2020\_G2-M[GSEA]

|  || Dataset | state43017 |
| Phenotype | NoPhenotypeAvailable |
| Upregulated in class | na\_pos |
| GeneSet | FELDMAN\_2020\_G2-M |
| Enrichment Score (ES) | 0.59010077 |
| Normalized Enrichment Score (NES) | 3.6443374 |
| Nominal p-value | 0.0 |
| FDR q-value | 0.0 |
| FWER p-Value | 0.0 |
Table: GSEA Results Summary

  

Fig 1: Enrichment plot: FELDMAN\_2020\_G2-M      
 Profile of the Running ES Score & Positions of GeneSet Members on the Rank Ordered List

  

| PROBE | GENE SYMBOL | GENE\_TITLE | RANK IN GENE LIST | RANK METRIC SCORE | RUNNING ES | CORE ENRICHMENT || 1 | TOP2A |  |  | 3 | 1.079 | 0.0092 | Yes |
| 2 | RRM2 |  |  | 7 | 0.875 | 0.0157 | Yes |
| 3 | SERPINE1 |  |  | 8 | 0.873 | 0.0272 | Yes |
| 4 | UBE2C |  |  | 11 | 0.802 | 0.0344 | Yes |
| 5 | TPX2 |  |  | 12 | 0.801 | 0.0449 | Yes |
| 6 | NUSAP1 |  |  | 13 | 0.801 | 0.0554 | Yes |
| 7 | HMGB2 |  |  | 14 | 0.786 | 0.0657 | Yes |
| 8 | FBXO5 |  |  | 15 | 0.785 | 0.0760 | Yes |
| 9 | NCAPG |  |  | 16 | 0.778 | 0.0862 | Yes |
| 10 | SPC25 |  |  | 17 | 0.763 | 0.0963 | Yes |
| 11 | PRC1 |  |  | 18 | 0.756 | 0.1062 | Yes |
| 12 | CDCA2 |  |  | 19 | 0.751 | 0.1160 | Yes |
| 13 | ESCO2 |  |  | 20 | 0.742 | 0.1258 | Yes |
| 14 | TACC3 |  |  | 21 | 0.741 | 0.1355 | Yes |
| 15 | GTSE1 |  |  | 22 | 0.732 | 0.1451 | Yes |
| 16 | HJURP |  |  | 23 | 0.731 | 0.1547 | Yes |
| 17 | KIF23 |  |  | 24 | 0.721 | 0.1641 | Yes |
| 18 | ASPM |  |  | 26 | 0.695 | 0.1716 | Yes |
| 19 | NDC80 |  |  | 27 | 0.695 | 0.1807 | Yes |
| 20 | ARHGAP11A |  |  | 30 | 0.683 | 0.1864 | Yes |
| 21 | KPNA2 |  |  | 33 | 0.676 | 0.1919 | Yes |
| 22 | CENPF |  |  | 34 | 0.670 | 0.2007 | Yes |
| 23 | KIF4A |  |  | 37 | 0.662 | 0.2061 | Yes |
| 24 | ANLN |  |  | 39 | 0.659 | 0.2131 | Yes |
| 25 | SMC4 |  |  | 41 | 0.655 | 0.2201 | Yes |
| 26 | MELK |  |  | 42 | 0.655 | 0.2287 | Yes |
| 27 | CENPE |  |  | 43 | 0.654 | 0.2372 | Yes |
| 28 | NUF2 |  |  | 44 | 0.651 | 0.2458 | Yes |
| 29 | PRR11 |  |  | 45 | 0.649 | 0.2543 | Yes |
| 30 | DLGAP5 |  |  | 46 | 0.647 | 0.2628 | Yes |
| 31 | KIFC1 |  |  | 47 | 0.645 | 0.2713 | Yes |
| 32 | KIF18A |  |  | 48 | 0.639 | 0.2796 | Yes |
| 33 | CENPA |  |  | 51 | 0.636 | 0.2847 | Yes |
| 34 | CKAP2 |  |  | 53 | 0.628 | 0.2913 | Yes |
| 35 | CCNA2 |  |  | 54 | 0.626 | 0.2995 | Yes |
| 36 | AURKA |  |  | 55 | 0.617 | 0.3076 | Yes |
| 37 | CKAP2L |  |  | 59 | 0.611 | 0.3106 | Yes |
| 38 | CENPK |  |  | 62 | 0.603 | 0.3153 | Yes |
| 39 | BUB1B |  |  | 64 | 0.596 | 0.3214 | Yes |
| 40 | CCDC34 |  |  | 65 | 0.595 | 0.3292 | Yes |
| 41 | KIF2C |  |  | 66 | 0.591 | 0.3370 | Yes |
| 42 | IGFBP5 |  |  | 67 | 0.590 | 0.3447 | Yes |
| 43 | SMC2 |  |  | 68 | 0.586 | 0.3524 | Yes |
| 44 | CDK1 |  |  | 69 | 0.585 | 0.3601 | Yes |
| 45 | H2AFX |  |  | 71 | 0.576 | 0.3660 | Yes |
| 46 | CKS1B |  |  | 72 | 0.575 | 0.3735 | Yes |
| 47 | RACGAP1 |  |  | 75 | 0.572 | 0.3777 | Yes |
| 48 | FOXM1 |  |  | 76 | 0.570 | 0.3852 | Yes |
| 49 | MAD2L1 |  |  | 77 | 0.569 | 0.3926 | Yes |
| 50 | PLK1 |  |  | 78 | 0.567 | 0.4001 | Yes |
| 51 | DBF4 |  |  | 79 | 0.567 | 0.4075 | Yes |
| 52 | CTGF |  |  | 81 | 0.564 | 0.4133 | Yes |
| 53 | CDCA8 |  |  | 82 | 0.562 | 0.4206 | Yes |
| 54 | CKS2 |  |  | 83 | 0.558 | 0.4280 | Yes |
| 55 | ECT2 |  |  | 84 | 0.558 | 0.4353 | Yes |
| 56 | KIF14 |  |  | 85 | 0.557 | 0.4426 | Yes |
| 57 | UBE2S |  |  | 87 | 0.555 | 0.4482 | Yes |
| 58 | AURKB |  |  | 89 | 0.548 | 0.4538 | Yes |
| 59 | PIF1 |  |  | 90 | 0.545 | 0.4609 | Yes |
| 60 | SPC24 |  |  | 91 | 0.544 | 0.4681 | Yes |
| 61 | CENPM |  |  | 92 | 0.544 | 0.4752 | Yes |
| 62 | CCNB1 |  |  | 93 | 0.544 | 0.4823 | Yes |
| 63 | PBK |  |  | 95 | 0.540 | 0.4878 | Yes |
| 64 | DEPDC1 |  |  | 97 | 0.536 | 0.4931 | Yes |
| 65 | SKA3 |  |  | 99 | 0.525 | 0.4984 | Yes |
| 66 | TMEM106C |  |  | 100 | 0.523 | 0.5052 | Yes |
| 67 | MXD3 |  |  | 104 | 0.519 | 0.5071 | Yes |
| 68 | CMC2 |  |  | 105 | 0.516 | 0.5139 | Yes |
| 69 | CDKN3 |  |  | 108 | 0.513 | 0.5173 | Yes |
| 70 | UBE2T |  |  | 110 | 0.512 | 0.5224 | Yes |
| 71 | CDCA3 |  |  | 111 | 0.511 | 0.5291 | Yes |
| 72 | DIAPH3 |  |  | 112 | 0.510 | 0.5358 | Yes |
| 73 | BUB1 |  |  | 113 | 0.508 | 0.5424 | Yes |
| 74 | TUBB4B |  |  | 114 | 0.507 | 0.5491 | Yes |
| 75 | RAD21 |  |  | 115 | 0.506 | 0.5557 | Yes |
| 76 | BIRC5 |  |  | 116 | 0.504 | 0.5623 | Yes |
| 77 | DTYMK |  |  | 118 | 0.503 | 0.5673 | Yes |
| 78 | SPDL1 |  |  | 126 | 0.492 | 0.5622 | Yes |
| 79 | TMPO |  |  | 127 | 0.492 | 0.5686 | Yes |
| 80 | SHCBP1 |  |  | 133 | 0.484 | 0.5667 | Yes |
| 81 | GMNN |  |  | 135 | 0.484 | 0.5714 | Yes |
| 82 | NCAPD2 |  |  | 136 | 0.483 | 0.5778 | Yes |
| 83 | TTK |  |  | 137 | 0.482 | 0.5841 | Yes |
| 84 | KIF22 |  |  | 152 | 0.459 | 0.5670 | Yes |
| 85 | KNSTRN |  |  | 155 | 0.456 | 0.5697 | Yes |
| 86 | SPAG5 |  |  | 156 | 0.455 | 0.5757 | Yes |
| 87 | CDKN2C |  |  | 164 | 0.445 | 0.5699 | Yes |
| 88 | TK1 |  |  | 167 | 0.444 | 0.5725 | Yes |
| 89 | DEPDC1B |  |  | 177 | 0.436 | 0.5633 | Yes |
| 90 | MKI67 |  |  | 179 | 0.436 | 0.5674 | Yes |
| 91 | RHNO1 |  |  | 183 | 0.432 | 0.5681 | Yes |
| 92 | CDC20 |  |  | 184 | 0.431 | 0.5738 | Yes |
| 93 | MIS18BP1 |  |  | 186 | 0.430 | 0.5778 | Yes |
| 94 | HMMR |  |  | 191 | 0.426 | 0.5767 | Yes |
| 95 | CKAP5 |  |  | 192 | 0.426 | 0.5823 | Yes |
| 96 | NEK2 |  |  | 194 | 0.423 | 0.5862 | Yes |
| 97 | MIS18A |  |  | 196 | 0.421 | 0.5901 | Yes |
| 98 | ZWINT |  |  | 212 | 0.407 | 0.5707 | No |
| 99 | H2AFZ |  |  | 216 | 0.405 | 0.5711 | No |
| 100 | POC1A |  |  | 234 | 0.390 | 0.5481 | No |
| 101 | PARPBP |  |  | 240 | 0.387 | 0.5449 | No |
| 102 | TRIP13 |  |  | 241 | 0.387 | 0.5500 | No |
| 103 | HMGN2 |  |  | 243 | 0.386 | 0.5534 | No |
| 104 | RTKN2 |  |  | 248 | 0.379 | 0.5518 | No |
| 105 | CCNB2 |  |  | 250 | 0.379 | 0.5551 | No |
| 106 | MPHOSPH9 |  |  | 254 | 0.375 | 0.5551 | No |
| 107 | GAS2L3 |  |  | 262 | 0.369 | 0.5484 | No |
| 108 | TUBB |  |  | 263 | 0.368 | 0.5532 | No |
| 109 | CENPW |  |  | 267 | 0.366 | 0.5531 | No |
| 110 | TUBG1 |  |  | 268 | 0.365 | 0.5579 | No |
| 111 | ANP32E |  |  | 280 | 0.358 | 0.5444 | No |
| 112 | CDC25B |  |  | 286 | 0.356 | 0.5408 | No |
| 113 | CALM2 |  |  | 290 | 0.354 | 0.5405 | No |
| 114 | DCBLD2 |  |  | 294 | 0.351 | 0.5402 | No |
| 115 | CENPN |  |  | 309 | 0.343 | 0.5216 | No |
| 116 | OIP5 |  |  | 315 | 0.341 | 0.5178 | No |
| 117 | DEK |  |  | 316 | 0.341 | 0.5223 | No |
| 118 | PHF19 |  |  | 319 | 0.339 | 0.5234 | No |
| 119 | EIF5 |  |  | 346 | 0.327 | 0.4848 | No |
| 120 | SPARC |  |  | 350 | 0.325 | 0.4841 | No |
| 121 | TMEM237 |  |  | 353 | 0.324 | 0.4851 | No |
| 122 | LMNB1 |  |  | 363 | 0.321 | 0.4744 | No |
| 123 | KIF20A |  |  | 392 | 0.311 | 0.4323 | No |
| 124 | RHEB |  |  | 404 | 0.307 | 0.4182 | No |
| 125 | LRR1 |  |  | 410 | 0.304 | 0.4139 | No |
| 126 | PTTG1 |  |  | 413 | 0.302 | 0.4146 | No |
| 127 | FABP7 |  |  | 414 | 0.302 | 0.4185 | No |
| 128 | C12orf75 |  |  | 433 | 0.296 | 0.3927 | No |
| 129 | MEST |  |  | 441 | 0.294 | 0.3850 | No |
| 130 | PRPSAP1 |  |  | 442 | 0.294 | 0.3889 | No |
| 131 | G2E3 |  |  | 446 | 0.294 | 0.3878 | No |
| 132 | MZT1 |  |  | 456 | 0.291 | 0.3768 | No |
| 133 | SAE1 |  |  | 463 | 0.289 | 0.3706 | No |
| 134 | TTYH1 |  |  | 469 | 0.287 | 0.3662 | No |
| 135 | RNASEH2A |  |  | 478 | 0.285 | 0.3567 | No |
| 136 | NUCKS1 |  |  | 483 | 0.285 | 0.3538 | No |
| 137 | CENPH |  |  | 501 | 0.281 | 0.3295 | No |
| 138 | PSMC3 |  |  | 506 | 0.280 | 0.3265 | No |
| 139 | TNFAIP8L1 |  |  | 510 | 0.279 | 0.3253 | No |
| 140 | FAM83D |  |  | 511 | 0.279 | 0.3289 | No |
| 141 | IFITM3 |  |  | 512 | 0.279 | 0.3326 | No |
| 142 | CDC27 |  |  | 520 | 0.278 | 0.3247 | No |
| 143 | LSM3 |  |  | 522 | 0.277 | 0.3266 | No |
| 144 | GGH |  |  | 564 | 0.270 | 0.2625 | No |
| 145 | RPA3 |  |  | 584 | 0.267 | 0.2347 | No |
| 146 | CCDC18 |  |  | 586 | 0.267 | 0.2365 | No |
| 147 | EXOSC8 |  |  | 647 | 0.256 | 0.1409 | No |
| 148 | METRN |  |  | 695 | -0.273 | 0.0669 | No |
| 149 | RPL31 |  |  | 705 | -0.284 | 0.0558 | No |
| 150 | SOX4 |  |  | 712 | -0.305 | 0.0499 | No |
| 151 | PRDX1 |  |  | 719 | -0.326 | 0.0443 | No |
| 152 | FGFBP3 |  |  | 721 | -0.334 | 0.0470 | No |
| 153 | BTG1 |  |  | 741 | -0.423 | 0.0212 | No |
| 154 | HES6 |  |  | 752 | -0.523 | 0.0116 | No |
Table: GSEA details [plain text format]

  

Fig 2: FELDMAN\_2020\_G2-M: Random ES distribution      
 Gene set null distribution of ES for **FELDMAN\_2020\_G2-M**
